# Application of QuEChERS extraction and LC–MS/MS for determination of pharmaceuticals in sewage sludges sampled across the Czech Republic

**DOI:** 10.1007/s11356-024-35508-8

**Published:** 2024-11-09

**Authors:** Pavlína Landová, Ludmila Mravcová, Šárka Poláková, Petra Kosubová

**Affiliations:** 1 Faculty of Chemistry, Brno University of Technology, Purkyňova 464/118, 612 00 Brno, Czech Republic; 2 Central Institute for Supervising and Testing in Agriculture (CISTA), Hroznová 63/2, 603 00 Brno, Czech Republic

**Keywords:** Pharmaceuticals, Micropollutants, Sewage sludge, QuEChERS, LC–MS/MS

## Abstract

The use of pharmaceuticals entails a significant risk of environmental contamination. Wastewater treatment plants (WWTPs) are considered to be the main contributors to contamination as they ineffectively eliminate these compounds from wastewater. Simultaneously, they produce solid waste, sludge, which often contains a variety of retained pollutants, including pharmaceuticals. Since sewage sludge is frequently applied to agricultural soil due to its rich nutrient content, pollutants are introduced into the environment in this way. Only a few studies have been carried out on the topic of the analysis of pharmaceuticals in sludge. Therefore, information on the occurrence of pharmaceuticals in sludge is limited. The present study employed quick, easy, cheap, effective, rugged and safe (QuEChERS) extraction and liquid chromatography coupled with tandem mass spectrometry (LC–MS/MS) analysis to establish a simple and reliable procedure for determining 16 pharmaceuticals (antibiotics, anticonvulsants, antidepressants and β-blockers) in sewage sludge. The method has been thoroughly validated, and parameters such as linear range, accuracy, precision, matrix effects and detection and quantification limits were assessed. Our method achieved low limits of quantification (0.5–9.0 µg kg^−1^) and satisfactory recoveries (51–101%). Forty sludge samples from different WWTPs across the Czech Republic were analysed. Fourteen compounds were detected and quantified in most samples, with antidepressants having the highest detection frequency and overall content. Sertraline, with a mean concentration of 521.0 µg kg^−1^, was notably prevalent alongside its metabolite norsertraline (mean concentration 204.9 µg kg^−1^). The antibiotic azithromycin was also found at higher levels (mean concentration 185.1 µg kg^−1^).

**Supplementary Information:**

The online version contains supplementary material available at 10.1007/s11356-024-35508-8.

## Introduction

The global consumption of pharmaceuticals is continuously increasing. In particular, medications intended to manage lifestyle-related conditions, including antihypertensives, lipid-modifying agents and antidiabetics, are increasingly used. Of equal significance is the increasing prevalence of antidepressant prescriptions, which is likely attributable to improved recognition of depression (González Peña et al. 2021). Consumption of antibiotics, which play a key role in treating bacterial infections, is also non-negligible (Browne et al. 2021).

The production and use of pharmaceuticals lead to environmental contamination, stemming from various sources. Notably, wastewater treatment plants (WWTPs) are considered the primary source of environmental contamination with these substances due to their limited removal efficacy (Anand et al. 2022). Furthermore, the wastewater treatment processes inevitably involve the production of solid waste, known as sewage sludge, which is often reused in agriculture as a soil amendment due to its rich nutrient content. For instance, in the Czech Republic in 2020, direct agricultural use amounted to almost 39% and composting accounted for 42% of the total waste disposal methods. A total of 219.11 thousand tonnes of sewage sludge were produced during this period (Eurostat 2024). Apart from plant growth-promoting nutrients (Eid et al. 2019, 2021; Swain et al. 2021), sewage sludge may also harbour a variety of contaminants, including heavy metals, polycyclic aromatic hydrocarbons, polychlorinated biphenyls, poly- and per-fluorinated compounds and microbial contamination (Souza et al. 2024). Many contaminants are now regulated by legislation (country dependent), with maximum allowable limits set (Hudcová et al. 2019). Exceeding these limits excludes the application of such sludges on agricultural soil.

The investigation of pharmaceuticals’ presence in wastewater and the effectiveness of their removal is a well-explored field of scientific research (Bijlsma et al. 2021; Eniola et al. 2022; Rodríguez-Serin et al. 2022; khalidi-idrissi et al. 2023). Noticeably, less information is available regarding the occurrence of pharmaceuticals in the sludge. The sludge matrix is much more complex; therefore, the analysis of pharmaceuticals within it is notably more intricate and demanding. However, several studies have been conducted on this topic, all of which reported positive findings for some of the target pharmaceuticals (Radjenović et al. 2009; Peysson and Vulliet 2013; Gago-Ferrero et al. 2015).

The consumption and introduction of pharmaceuticals into the environment may cause several issues. Notably, the use of antibiotics is associated with the development of antimicrobial resistance (AMR), a significant health concern recognised by the World Health Organization (Browne et al. 2021). Additionally, AMR is worsened by the misuse of antibiotics, inappropriate treatment approaches and their overuse in animal farming (Manyi-Loh et al. 2018). Furthermore, the adverse effects of various pharmaceuticals on the soil microbial communities (Pino-Otín et al. 2017; Wu et al. 2021) and their adverse impact on aquatic ecosystems cannot be ruled out (Hossain et al. 2019). It is also confirmed that pharmaceuticals are transferred into edible crops from reclaimed wastewater (Ben Mordechay et al. 2021; Kodešová et al. 2024). Additionally, runoff and leaching from agricultural fields, irrigated by reclaimed wastewater or amended by sewage sludge, can transport these compounds to the surface and groundwaters (Petrović et al. 2014). Considering these aspects, it is evident that there is a particular risk for human exposure, e.g. via unintentional intake through contaminated crops (Holling et al. 2012; Ben Mordechay et al. 2021) or contaminated drinking water (Nováková et al. 2023). To evaluate environmental and human health risks related to sludge production and management, it is desirable to explore further the presence of various pharmaceuticals and other micropollutants in sludge. Such vital information can be a part of the knowledge needed for the possible implementation of regulatory policies regarding sludge disposal and reuse management.

The determination of pharmaceuticals in solid samples was previously accomplished through solvent extraction employing ultrasound (USE) (Gago-Ferrero et al. 2015; Golovko et al. 2016) or pressurised solvent extraction (PLE) (Radjenović et al. 2009), followed by pre-concentration and/or additional purification steps, including offline or online solid-phase extraction (SPE) (Riva et al. 2021), dispersive solid-phase extraction (D-SPE) or solvent–solvent extraction (Peysson and Vulliet 2013). QuEChERS (an acronym for quick, easy, cheap, effective, rugged and safe) extraction was initially designed to analyse pesticides in fruits and vegetables (Anastassiades et al. 2003). Over the years, this technique has become increasingly popular, and its use is now being tested to analyse other types of chemicals and matrices (Bruzzoniti et al. 2014). Its applicability has been confirmed for analysing pesticides in soils (Kosubová et al. 2020), sediments (Nannou et al. 2018) and drinking water treatment sludge (Cerqueira et al. 2014). The use of QuEChERS for analysing pharmaceuticals in sludges and other environmental matrices is gaining attention, and more articles on this topic are becoming available (Peysson and Vulliet 2013; De Mastro et al. 2022; Miserli et al. 2023). Liquid chromatography coupled with tandem mass spectrometry (LC–MS/MS) technique is commonly the preferred method for ultimate quantification, primarily due to its compatibility with the chemical properties of pharmaceuticals and its notable sensitivity and selectivity of mass spectrometric analysers. This heightened sensitivity and selectivity are essential when analysing compounds at trace levels (in the parts per billion ranges) within complex environmental samples.

The main objective of this study was to employ QuEChERS extraction in conjunction with LC–MS/MS for the comprehensive analysis of 16 pharmaceutical compounds and their metabolites in sludge, with the main emphasis placed on the simplicity of the procedure and broad applicability to sludges with variable composition. Compounds from the groups of antibiotics, anticonvulsants, antidepressants and β-blockers were targeted because of their relevance in terms of consumption and known ecotoxicological significance. The developed method was subsequently used to screen pharmaceuticals in forty distinct sludge samples from various locations across the Czech Republic. The obtained data provide representative insights into the presence of the target analytes within the sludge. To the best of our knowledge, such a study covering this large sample set is currently unavailable in the literature. In addition, information regarding the analysis and presence of some listed compounds in sewage sludge is absent or relatively scarce.

## Materials and methods

### Chemicals and reagents

All native and isotopically labelled internal standards of pharmaceuticals employed were of ≥ 95% purity. Bisoprolol, carbamazepine-10,11-epoxide, sertraline hydrochloride, fluoxetine hydrochloride, azithromycin, carbamazepine, clarithromycin, trimethoprim, acebutolol hydrochloride, atenolol, paroxetine hydrochloride hemihydrate, citalopram hydrobromide, propranolol hydrochloride, metoprolol tartrate, sulfamethoxazole, ( ±)-sertraline-D3 hydrochloride (100 µg mL^−1^ in methanol), fluoxetine-D5 (1 mg mL^−1^ in methanol), carbamazepine-D10 (100 µg mL^−1^ in methanol), trimethoprim-D9, atenolol-D7 and ( ±)-propranolol-D7 (100 µg mL^−1^ in methanol) were purchased from Sigma-Aldrich (Steinheim, Germany). Rac-norsertraline hydrochloride from LGC standards (Luckenwalde, Germany), sulfamethoxazole-D4 (100 µg mL^−1^ in methanol) from Neochema (Bodenheim, Germany) and clarithromycin-N-methyl-D3 and azithromycin-D3 from TRC (Toronto, Canada).

The HPLC-grade solvents (methanol and acetonitrile) were purchased from Sigma-Aldrich (Saint Louis, USA). LC–MS grade formic acid (LiChropur®) and methanol (LiChrosolv® Hypergrade) were purchased from Merck (Darmstadt, Germany). Deionised water (ultra-pure water, 18 MΩ cm^−1^) was obtained using the purification system from Stakpure (Niederahr, Germany). The QuEChERS buffering salt kit was prepared in-house. It consisted of 4 g anhydrous magnesium sulphate, 1 g sodium chloride (both annealed at 550 °C in a muffle furnace for 6 h), 0.5 g disodium citrate sesquihydrate and 1 g trisodium citrate dihydrate. Nylon filters with a 0.22-µm pore size and 13 mm diameter were acquired from Labicom (Olomouc, Czech Republic).

Stock solutions of the native pharmaceuticals were prepared at a concentration of 1 mg mL^−1^ in methanol and stored in amber glass vials in a refrigerator at 4 °C protected from solvent evaporation. The working mix solutions were prepared by diluting the stock solutions with methanol, and they were subsequently stored in amber glass vials in a freezer at − 20 °C. Calibration solutions were prepared fresh daily. Purchased solutions of isotopically labelled internal standards (ILIS) were kept in the freezer protected from light and solvent evaporation. ILIS purchased in the form of powders were prepared and stored in the same manner as native pharmaceuticals. Individual ILIS were mixed and diluted to a concentration of 0.5 µg mL^−1^ and stored in amber glass vials in a freezer at − 20 °C.

### Origin of samples, sampling and sample preparation

Sludges were collected from forty municipal wastewater treatment plants across the Czech Republic between February and June 2020. The sampling campaign specifically targeted those facilities where a portion, or the entire sludge production, is subsequently used for direct application to agricultural soil or composting (more information regarding the sampling is available in the Supplementary material, Table S1).

Sampling and sample preparation procedures were performed by qualified personnel following standard protocols. In brief, stabilised dewatered sludge from containers or heaps was sampled using a shovel or slot drill to obtain a representative sample of approximately 1 kg. The samples were packed into PE bags and transported to the laboratory in cooling boxes. Subsequently, they were sanitised using gamma irradiation, dried, milled, sieved using a 1-mm mesh and stored in the dark and cold until analysis.

### Analytical procedures

#### Extraction

The QuEChERS technique was employed to extract target pharmaceuticals from the sludge. 2 g (dry weight) of sludge was combined with 10 mL of deionised water and 10 mL of acetonitrile in a 50-mL polypropylene Falcon tube. The mixture was subjected to extraction for 15 min on a vertical mechanical shaker Geno Grinder 2010 (SPEX SamplePrep, Metuchen, USA), operating at 500 strokes min^−1^. After the initial extraction, a cold ceramic homogeniser and QuEChERS salts were added to the sample. The tube was sealed and vigorously shaken for 5 s. Subsequently, the tubes were placed in an ice bath for 5 min to dissipate the excess heat generated by salt addition. This was followed by an additional 2-min mechanical shaking at 500 strokes min^−1^. The tubes were then centrifuged for 5 min at 5000 rpm. The resulting supernatant was carefully transferred into a clean 15-mL Falcon tube and stored in a freezer until analysis.

The preparation of extracts for LC–MS/MS analysis was conducted as follows: a 500-µL portion of the sample extract was combined with 10 µL of a 5% formic acid solution in acetonitrile and 10 µL of ILIS mixture containing nine internal standards (resulting concentration of 5 ng mL^−1^ or 50 µg kg^−1^); the volume was then adjusted to 1000 µL with deionised water. If needed, another dilution was prepared, where only 100 µL of the sample extract was mixed with 400 µL of acetonitrile, 10 µL of 5% formic acid and 10 µL of the ILIS mixture. The volume was adjusted to 1000 µL with deionised water. The prepared samples were vortexed and filtered through 0.22-µm nylon syringe filters into chromatographic vials. The LC–MS/MS analysis was then performed.

#### Analytical determination

Chromatographic separation was performed on an Infinity 1290 binary pump liquid chromatograph (Agilent, Santa Clara, USA) using a Kinetex Biphenyl column (Phenomenex, Torrance, USA) with dimensions of 2.1 mm width, 100 mm length and 2.6 µm particle size. A mass spectrometer QTrap 4500 (AB Sciex, Framingham, USA) was used for detection, employing positive electrospray ionisation and scheduled MRM (multiple reaction monitoring) mode for data acquisition.

Mobile phase A consisted of MQ water with 0.1% formic acid (v/v), whereas mobile phase B comprised methanol with 0.1% formic acid (v/v). The injection volume was set at 1 µL, the column temperature was maintained at 40 °C and the flow rate was set to 0.5 mL min^−1^. The gradient programme was defined as follows: from 0 to 3 min, the composition of B increased from 5 to 95%. These conditions were sustained until 6.2 min when the composition reverted from 95% B to 5% B within 6.3 min. The total analysis time was 8.5 min. The mass spectrometric settings were as follows: an ion source temperature of 475 °C, an ion spray voltage of 4500 V, a curtain gas set at 40 psi (nitrogen), ion source gas 1 and ion source gas 2, both set at 45 psi (air) and the collision gas (nitrogen) adjusted to a medium value. Further details regarding the mass transitions are provided in Table S2. Data acquisition was performed using Analyst® software version 1.7.1 (AB Sciex, Framingham, USA), and for data processing, the software SciexOS version 2.0 (AB Sciex, Framingham, USA) was used.

Quantification was conducted using internal standards, with details concerning their assignment to native analytes available in Table S2. The presence of compounds in the samples was confirmed based on various criteria, including a retention time deviation of a maximum of ± 0.1 min and ion ratio within the limits of ± 30%.

## Results and discussion

### Instrumental method optimisation

Chromatographic separation of pharmaceuticals is mainly performed on various C18 columns (Meng et al. 2021). Given the availability of a phenyl-based stationary phase column, known for its favourable chemistry in separating compounds with aromatic ring structures and enhancing the retention of polar analytes, we opted to evaluate this column. The selection of methanol as the organic phase was evident because acetonitrile hinders the π-π interactions between the stationary phase and the separated compounds. As a pH modifier, formic acid was chosen and added to both phases to reach a content of 0.1%. Subsequently, parameters such as the gradient programme, flow rate, column temperature and injection volume were optimised. Since the column provided good results regarding peak shapes and retention characteristics, there was no need to make further changes in the mobile phase composition or use another type of column chemistry.

The MRM mass transitions used in the MS/MS method were derived from our previous study (Niemi et al. 2022). They were also adopted from the literature (Gros et al. 2012; Grabic et al. 2012; Gago-Ferrero et al. 2015). Additionally, some transitions were obtained experimentally through the direct injection of solvent standards into the mass spectrometer. All compounds were analysed in positive ionisation mode using the ions [M + H]^+^ as precursors, except for azithromycin, for which the double-charged ion [M + 2H]^2+^ was more favourable. A comprehensive set of optimised MRM transitions was created, ideally exceeding two MRMs per compound. The most specific and sensitive MRM transitions were selected based on the analysis of several matrix standards prepared from extracts of sludge samples.

Furthermore, ion source parameters such as source temperature and flow rate of gases were adjusted to achieve optimal responses for all analytes. Before the final estimation of detection limits, adding of a small amount of formic acid into the vial was adopted from the literature to increase the responses of some analytes (Gago-Ferrero et al. 2015). Satisfactory quantification limits in units of micrograms per kilogram have been estimated, even when using a low injection volume of 1 µL. Using a low injection volume is particularly advantageous for analysing complex samples as the introduction of matrix co-extracts into the instrument is kept as low as possible.

### QuEChERS extraction

An extraction procedure based on the EN 15662:2018 standard involving citrate buffering was applied. This method has also been proven effective in determining a wide range of pesticides in soils (Kosubová et al. 2020). Because a blank sludge matrix was unavailable, real sludge samples were used for optimisation. Sludges’ composition can vary considerably (Sichler et al. 2022) depending on their origin and other factors. Such variations can affect analyte behaviour during sample preparation and analysis. Considering these aspects, the individual optimisation steps were always conducted on at least two distinct sludge samples. Samples for optimisation were selected based on a comparison of their known properties, such as pH, organic matter content and elemental composition (Fig. S1).

The first recovery trial was performed using four samples, each spiked with a mixture of native pharmaceuticals to attain a concentration of 200 µg kg^−1^. The spiked samples were subjected to vortexing and allowed to settle for an additional 30 min to facilitate solvent evaporation and analyte binding to the material. The procedure was conducted as described in the Materials and methods. Extraction recoveries were calculated by comparing the determined concentrations in spiked samples with those obtained from post-extraction matrix standards prepared from the non-spiked sample extracts processed parallel to spikes.

**Fig. 1:**
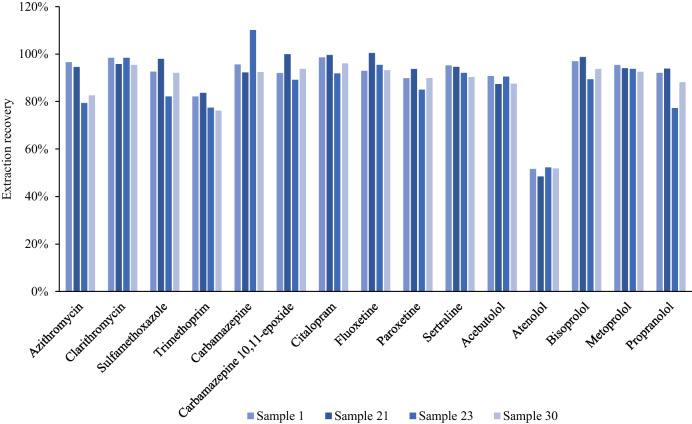
Extraction recoveries of pharmaceuticals obtained in the initial testing stage in four different sewage sludge samples at a fortification level of 200 µg kg^−1^

As shown in Fig. 1, the initial test confirmed that the proposed extraction procedure is well-suited for determining all target compounds. The average recoveries for individual compounds were deemed satisfactory, falling within the 51 to 98% range (median 91%), with relative standard deviations (RSDs) ranging from 1.3 to 9.7%. The β-blocker atenolol showed the lowest recovery, likely due to its higher polarity compared to the other tested compounds. The QuEChERS extraction is distinctive for its salting-out step, which separates the organic (acetonitrile) and water phase into two layers. While this step can be advantageous in simplifying the matrix composition of the resulting extract, it entails the risk of losing more polar compounds, as observed with atenolol.

### Dispersive-SPE cleanup

The sludge matrix yields complex extracts that can complicate the instrumental analysis, leading to signal suppression or enhancement, especially when LC–MS/MS with the electrospray ionisation technique is used. Various cleanup techniques, including dispersive solid-phase extraction (D-SPE) with distinct sorbents (Perestrelo et al. 2019), can be used to mitigate the complexity of these extracts. Two D-SPE mixtures were selected for cleanup testing (Peysson and Vulliet 2013; Bruzzoniti et al. 2014; Rashid et al. 2020). Mixture A comprised 50 mg of octa-decyl silica gel (ODS), 50 mg of primary secondary amine (PSA) and 300 mg of MgSO_4_, whereas mixture B comprised 300 mg of PSA and 300 mg of MgSO_4_. Graphitised carbon black (GCB), a widely used adsorbent known for retaining planar molecules such as pigments and sterols (Anastassiades et al. 2003), was excluded due to the well-documented strong interactions between GCB and azithromycin (Peysson and Vulliet 2013).

Cleanup was assessed on two distinct samples; each spiked to attain a concentration of 200 µg kg^−1^. Two millilitres of the extract were transferred into a 5-mL Falcon tube containing pre-weighed D-SPE mixture A or B. The tube was sealed, manually shaken for 2 min and centrifuged for 5 min at 4000 rpm. Subsequently, the processed extracts were prepared for instrumental analysis.

**Fig. 2:**
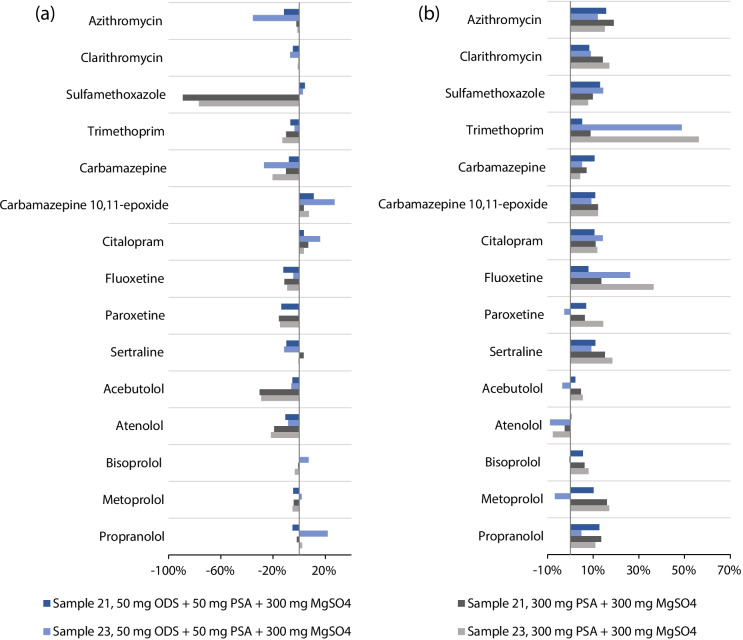
Differences in **a** extraction recoveries and **b** matrix effects with and without cleanup for all tested compounds in two different sewage sludge sample extracts using two D-SPE mixtures

The impact of this procedure was evaluated by calculating the differences between recoveries with and without cleanup. As shown in Fig. 2a, the cleanup mainly negatively affected the recovery. Significant declines were observed for sulfamethoxazole using mixture B, which contained a sixfold higher amount of PSA than mixture A. However, the azithromycin loss can be attributed solely to the ODS adsorbent present in mixture A. In addition to recoveries, differences in absolute (without using ILIS for correction) matrix effects (MEs) between extracts with and without cleanup were calculated. This assessment provided a straightforward means of evaluating the effectiveness of the cleanup procedure in reducing matrix co-extracts. MEs were calculated by comparing the analyte signals from the post-extraction and post-cleanup matrix standards with the response from the solvent standards. As depicted in Fig. 2b, the cleanup procedure generally reduced MEs (represented in positive values), with trimethoprim being the most affected compound. Nevertheless, the overall improvement of MEs averaged only 9% with mixture A or 13% with mixture B.

In conclusion, the above-described cleanup procedure did not prove to be effective and robust. The combination of analytes’ chemical properties and the sludge’s matrix composition may necessitate using different sorbents or an utterly different cleanup procedure to address this issue. Nevertheless, the instrumental method demonstrated sufficient sensitivity even when accounting for the anticipated absolute MEs. Thus, the cleanup step was omitted entirely for the sake of simplification of sample preparation and reduction of additional errors and costs. A set of internal standards was used to correct MEs, and its evaluation is provided in the following section of the “Method validation”.

### Method validation

The validation process included the evaluation of accuracy, precision, matrix effects, linearity and limits of detection and quantification, which are summarised in Table 1.

**Table 1:** Validation results for the determination of pharmaceuticals in sewage sludge, including the linear calibration range with the coefficients of determination (*r*^2^), method detection limits (MDL), method quantification limits (MQL) and recoveries with RSDs at the spiking levels of 10 µg kg^−1^ and 200 µg kg^−1^

Analyte	Calibration		MDL (µg kg^−1^)	MQL (µg kg^−1^)	Spiking level 10 µg kg^−1^			Spiking level 200 µg kg^−1^		
	Linear range (µg kg^−1^)	*r*^*2*^			Recovery (%)	RSD (%)	*n*	Recovery (%)	RSD (%)	*n*
Azithromycin	0.5–600	0.9985	1.3	4.0	n. a	n. a	n. a	81	11	29
Clarithromycin	0.5–1000	0.9994	0.7	2.0	93	12	6	98	8	39
Sulfamethoxazole	0.5–1000	0.9996	3.0	9.0	86	7	8	94	7	40
Trimethoprim	0.5–600	0.9991	0.2	0.5	72	6	8	76	6	40
Carbamazepine	0.5–400	0.9964	0.2	0.5	97	1	3	96	4	39
Carbamazepine 10,11-epoxide	0.5–600	0.9962	0.7	2.0	94	4	8	97	5	40
Citalopram	0.5–1000	0.9996	0.3	1.0	n. a	n. a	n. a	101	3	8
Fluoxetine	0.5–1000	0.9998	0.7	2.0	90	3	8	94	6	40
Paroxetine	0.5–1000	0.9997	1.3	4.0	93	7	8	83	8	40
Sertraline	0.5–1000	0.9993	0.3	1.0	n. a	n. a	n. a	92	9	6
Norsertraline	2–1000	0.9997	1.0	3.0	n. a	n. a	n. a	77	10	26
Acebutolol	0.5–1000	0.9996	1.3	4.0	89	4	8	87	6	40
Atenolol	0.5–1000	0.9992	1.3	4.0	52	16	8	51	7	40
Bisoprolol	0.5–1000	0.9995	0.7	2.0	90	2	8	95	5	40
Metoprolol	0.5–1000	0.9996	0.7	2.0	92	4	3	94	4	39
Propranolol	0.5–1000	0.9994	0.3	1.0	86	2	8	89	7	40

*n. a.* not assessed

Linearity was evaluated based on a seven-point calibration curve prepared from 0.5 to 1000 µg kg^−1^ (equivalent to 0.05 to 100 ng mL^−1^), with internal standards added at a concentration of 50 µg kg^−1^ (5 ng mL^−1^). A linear function with the 1/*x* weighing was used for all 16 analytes. The following criteria were assessed: the deviation of the back-calculated concentration from the actual concentration, which had to fall within the range of ≤  ± 20% at each calibration level, and the coefficient of determination (*r*^*2*^), which was required to be ≥ 0.99. Linearity across the entire calibration range was confirmed for most of the compounds.

Accuracy and precision were assessed at two fortification levels of 10 µg kg^−1^ and 200 µg kg^−1^. Given the reasons outlined in the preceding chapter, validation was conducted on real samples of forty different sludges. Applying this approach has become more challenging for some compounds due to their considerable content in samples. Analyte recovery data were excluded from evaluation when the content in the non-spiked sample reached the fortification level. Due to these issues, the fortification level of 10 µg kg^−1^ could not be validated for certain substances. The number of data points (*n*) used for calculating the recovery and RSD for each compound and fortification level is detailed in Table 1. The resulting recoveries at the level 200 µg kg^−1^ were consistent with those obtained during the optimisation, ranging from 51 to 101% with a median value of 93%. Even with the large and diverse set of samples, the RSDs remained within acceptable limits, with the highest RSD being 11% for azithromycin. At the fortification level of 10 µg kg^−1^, the recoveries ranged from 52 to 97% with a median value of 90%, and a maximum RSD of 16% was obtained for atenolol. Analytes recoveries from individual samples are provided in Table S3 and Table S4. As atenolol showed consistently lower recoveries, the concentrations determined in real samples must be corrected using an average recovery factor of 0.51.

**Fig. 3:**
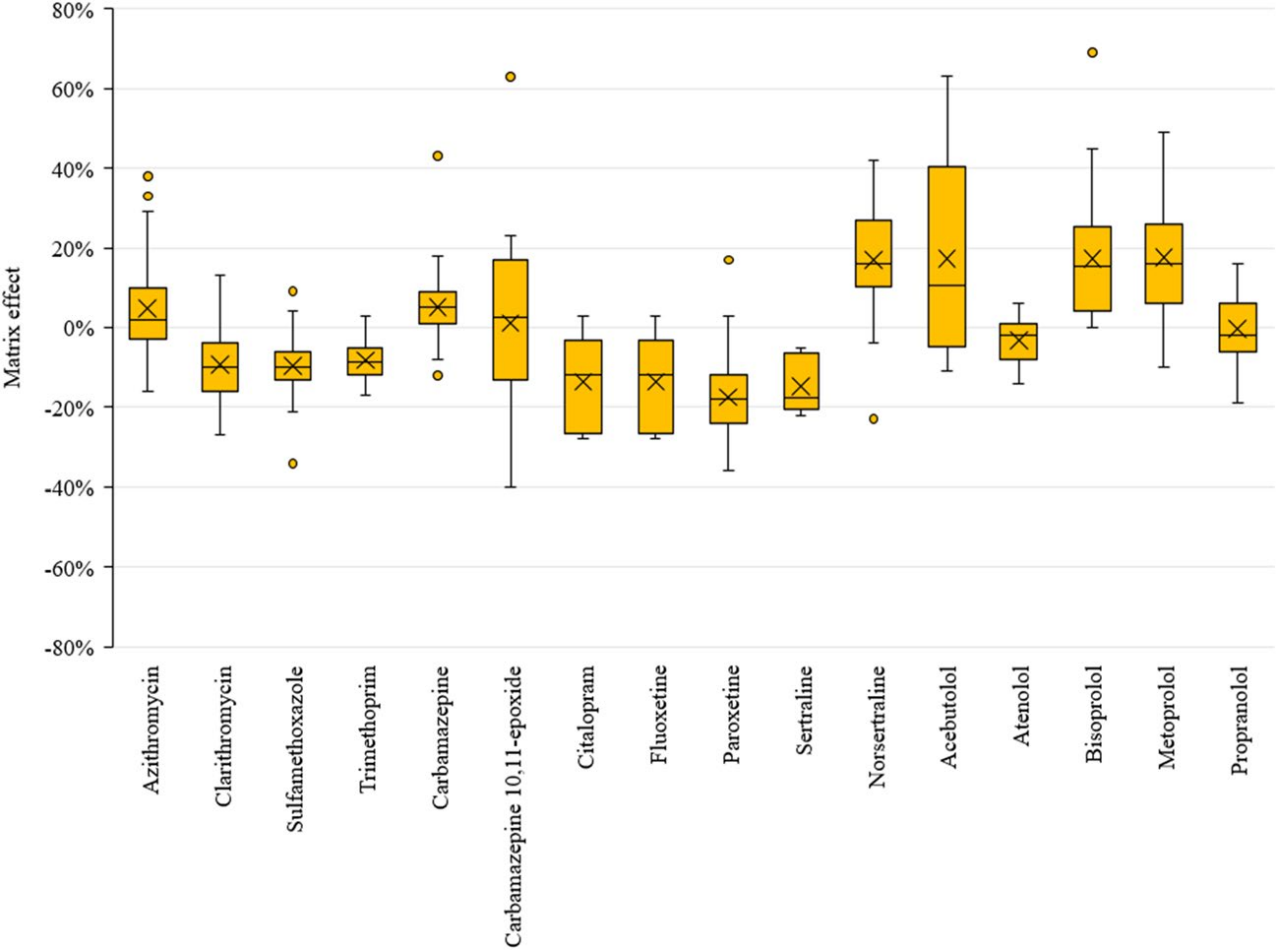
Box plot of matrix effects for all pharmaceuticals in all sludge samples

MEs (expressed as relative matrix effects with ILIS corrections) were assessed on post-extraction matrix standards prepared at 200 µg kg^−1^ concentration. The responses from the blank samples were subtracted from the responses of the matrix standards and then compared with those from solvent standards prepared at the same level. The results are depicted in Fig. 3; positive values represent signal enhancement, while negative values represent signal suppression. On average, MEs ranged within ± 20% for all compounds. However, they exhibited more significant variability from sample to sample for some analytes. Generally, better results were achieved for compounds having their isotopically labelled analogues as internal standards.

When internal standards failed to achieve ME within the acceptable range of ± 20%, corrections were made through either standard addition or further dilution of the extracts. The latter method, involving a tenfold dilution, proved to be the most practical and effective approach in mitigating the bias caused by ME (further information available in the Supplementary material, Fig. S2). However, routinely analysing only tenfold diluted extracts would require a more sensitive mass spectrometer to maintain low detection and quantification limits.

The method detection (MDL) and quantification (MQL) limits were estimated using a factor of 3 and 10 of standard deviation on the lowest calibration level, respectively. Other factors, such as a sufficient signal-to-noise ratio (S/N ≥ 3 for detection limits; S/N ≥ 10 for quantification limits), were also considered. The resulting values were additionally corrected for the worst-case scenario in terms of the absolute matrix effects, and in the case of atenolol, its lower recoveries were also considered. Most of the compounds had MQL in the range from 1.0 to 4.0 µg kg^−1^; the worst MQL of 9.0 µg kg^−1^ was obtained only for sulfamethoxazole, which suffered from low MS sensitivity in combination with excessive signal suppression due to matrix interferences. The obtained MQLs are usually lower than those previously reported in the literature (Table S5).

### Occurrence of pharmaceuticals in the sludge

Nearly all analytes were detected in each sludge sample, with antidepressants being the most frequently identified group of pharmaceuticals. Sulfamethoxazole was found only in three samples at levels around the detection limit. Only the carbamazepine metabolite carbamazepine-10,11-epoxide was not detected in any sample. The summary of results for individual compounds with the percentage of detection frequency, average, median, minimal and maximal contents found are listed in Table 2. Individual results are provided in Table S6.

**Table 2:** Detection frequency and the average, median, minimal and maximal determined concentrations of pharmaceuticals in the forty sewage sludge samples

	**% detection frequency (*n* = 40)**	**Concentration (µg kg**^−1^ **d. w.)**			
		**Average**	**Median**	**Minimum**	**Maximum**
**Antibiotics**					
Azithromycin	100	185.1	163.9	< 4.0	644.4
Clarithromycin	100	40.63	19.66	< 2.0	374.9
Sulfamethoxazole	8	< MDL	< MDL	< MDL	< 9.0
Trimethoprim	100	12.54	1.38	< 0.5	95.51
**Anticonvulsants and their metabolites**					
Carbamazepine	100	60.59	46.57	5.31	215.6
Carbamazepine-10,11-epoxide	0	< MDL	< MDL	< MDL	< MDL
**Antidepressants and their metabolites**					
Citalopram	100	382.4	369.1	65.31	977.1
Fluoxetine	100	18.97	14.05	3.06	91.50
Paroxetine	90	13.78	11.94	< MDL	39.48
Sertraline	100	521.0	488.7	163.4	1250
Norsertraline	100	204.9	174.1	44.83	749.9
**β-blockers**					
Acebutolol	98	20.27	11.92	< MDL	80.95
Atenolol	58	9.07	7.72	< MDL	46.57
Bisoprolol	98	13.44	11.83	< MDL	55.90
Metoprolol	100	90.57	77.66	5.10	596.1
Propranolol	85	4.99	4.31	< MDL	15.01

**Fig. 4:**
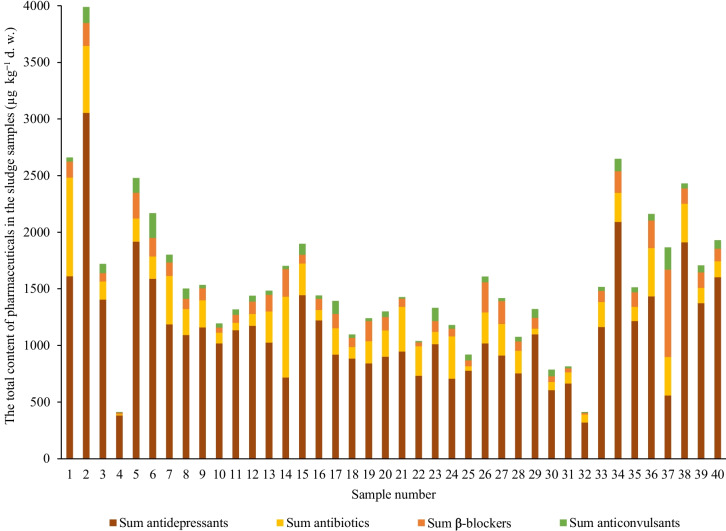
Total content of pharmaceuticals (stacked in groups) in individual sludge samples

The total quantities of target pharmaceuticals (sorted into their pharmacological groups) in individual samples are depicted in Fig. 4. Antidepressants, followed by antibiotics, dominated the overall composition. In only one sample (sample 37), the β-blockers prevailed over the other groups. The total content of pharmaceuticals in individual samples was relatively variable, ranging from 411 µg kg^−1^ in sample 4 to 3988 µg kg^−1^ in sample 2, with an average of 1570 µg kg^−1^. As the data are derived solely from a single episode of grab sampling at each WWTP, making comparisons and drawing conclusions between individual treatment plants is not feasible and was not the objective of this study. Numerous factors may influence the resulting concentrations, making such assessments challenging. Nevertheless, this comprehensive set of results may provide an indicative average concentration level for the analytes of interest in wastewater sludges in the Czech Republic.

The macrolide antibiotic azithromycin was detected at the highest median level of 163.9 µg kg^−1^, ranging from < MQL (4.0 µg kg^−1^) to 644.4 µg kg^−1^. The other macrolide representative, clarithromycin, was detected at an approximately eight times lower median level despite its higher consumption in the Czech Republic. The higher levels of azithromycin are most likely attributed to its higher potential for sorption onto sludge (Ben et al. 2018; Ajibola and Zwiener 2022). In addition, the differences can be caused by the different metabolisation and behaviour of these two substances in sewage systems. Trimethoprim was detected in all samples with concentrations ranging from < MQL (0.5 µg kg^−1^) to 95.51 µg kg^−1^. Trimethoprim is often combined with sulfamethoxazole, which was detected in only three samples at a level between its MDL and MQL (9.0 µg kg^−1^). Higher sulfamethoxazole MQL, different levels of metabolisation and degradation, or its lower affinity to sludge might explain this difference. Furthermore, both compounds are frequently detected in the water phase (in both influent and effluent of WWTPs) at considerable levels (Rossmann et al. 2014; Petrie et al. 2015; Ben et al. 2018). Thus, the sorption onto sludge for both compounds is most probably the minor elimination route during wastewater treatment. Anticonvulsant carbamazepine was detected in 100% of the samples, with concentrations ranging from 5.31 to 215.6 µg kg^−1^. Carbamazepine is known for undergoing extensive metabolisation in the human body, producing various metabolites, including, still pharmacologically active, carbamazepine-10,11-epoxide (Petrie et al. 2015). This metabolite was also included in this study. Nevertheless, its presence was not confirmed in any of the samples, possibly due to its higher polarity and subsequent degradation to other metabolites (Miao et al. 2005). Antidepressants were found at considerably higher levels than previously described compounds, most likely due to their high consumption (applies to sertraline and citalopram) in the Czech Republic and their lipophilic nature. Sertraline was present in 100% of the samples, with concentrations ranging from 163.4 to 1250 µg kg^−1^. Its major, still pharmacologically active metabolite, norsertraline, was detected at 44.83 to 749.9 µg kg^−1^ levels. Citalopram was present in all samples at concentrations ranging from 65.31 to 977.1 µg kg^−1^. Fluoxetine and paroxetine were also detected and quantified, although at lower concentrations in tens of micrograms per kilogram. β-blockers were detected in the following increasing order of percentage of positive results: atenolol, propranolol, acebutolol, bisoprolol and metoprolol. Their overall lower content is most likely attributed to their low sorption coefficients to sludge, as described in a previous study (Scheurer et al. 2010). Metoprolol was detected at approximately 6.5 times higher levels (77.66 to 596.1 µg kg^−1^) than bisoprolol (ND to 55.90 µg kg^−1^). Despite the negligible consumption of propranolol in the Czech Republic, its presence was confirmed in 85% of samples, with concentrations ranging from ND to 15.01 µg kg^−1^. Aspects such as different metabolisation and degradation rates and more lipophilic character might partly explain its content. In addition, this compound can be sold over the counter and abused to self-medicate panic and anxiety disorders (Kriikku et al. 2021).

A comparison of our findings with those of previous studies has its limitations. The consumption of individual medicines can vary between countries (Riva et al. 2021). Additionally, it can change over the years. Other aspects, such as seasonal effects (Aydın et al. 2022) and variable types of sludges analysed in the other studies, must be considered too. Furthermore, differences between quantification limits applied in individual studies can make the comparison difficult. Nevertheless, patterns in our findings are mainly similar to those observed in the literature (Table S5). Higher levels of azithromycin (60.8 to 267 µg kg^−1^) over clarithromycin (< MQL to 41.1 µg kg) were reported by Gago-Ferrero et al. (2015). Low concentrations (in units or tens of micrograms per kilogram) or findings below detection limits were usually reported for sulfamethoxazole and trimethoprim (Radjenović et al. 2009; Riva et al. 2021; Miserli et al. 2023). While carbamazepine has been quantified in sludges in several studies, ranging from 13.1 µg kg^−1^ (Miserli et al. 2023) to 258.1 µg kg^−1^ (Miao et al. 2005), the presence of its metabolite carbamazepine-10,11-epoxide has not been confirmed (Miao et al. 2005). Higher concentrations were observed for antidepressants, commonly in the tens to hundreds of micrograms per kilogram (Radjenović et al. 2009; Peysson and Vulliet 2013; Gago-Ferrero et al. 2015; Miserli et al. 2023). Our findings for citalopram, sertraline and norsertraline are, in most cases, considerably higher. As for β-blockers, less information regarding the occurrence of some of these compounds (acebutolol and bisoprolol) in sludges is currently available in the literature. In addition, more significant differences in reported concentrations of individual compounds were observed in this group. Maximal levels of 16.8 µg kg^−1^ were reported for metoprolol (Gago-Ferrero et al. 2015), while more lipophilic propranolol was quantified up to 849 µg kg^−1^ (Peysson and Vulliet 2013).

## Conclusions

In summary, we present a streamlined method for the determination of pharmaceuticals from different pharmacological classes in sewage sludge, utilizing QuEChERS extraction and LC–MS/MS determination. Its applicability was thoroughly verified in a large set of forty different samples. For most compounds, extraction recoveries were above 80%, and well-acceptable RSDs were achieved with a maximum value of 16% for atenolol. Low quantification limits were reached in most cases in the 1.0 to 4.0 µg kg^−1^ range.

More importantly, our study provides the original and valuable information about the content of these compounds in sludges in the Czech Republic. The analysis revealed the ubiquitous presence of almost all target compounds, with antidepressants being present at the highest levels, reaching up to 1250 µg kg^−1^ for sertraline, 977.1 µg kg^−1^ for citalopram and 749.9 µg kg^−1^ for norsertraline. Azithromycin was also quantified at considerable levels from the group of antibiotics, reaching up to 644.4 µg kg^−1^. Only carbamazepine-10,11-epoxide was not detected in any samples. Sulfamethoxazole was also sporadically present, with only 8% positive detection between its MDL and MQL. On average, the content of other compounds was in the order of tens of micrograms per kilogram. To the best of our knowledge, the occurrence of β-blocker acebutolol in sludges was reported for the first time.

The results of our study underline the importance of further research concerning the presence of pharmaceuticals in sludges, mainly due to the current, widely used disposal technique of direct application to agricultural soil. The impact of such application should be thoroughly examined, along with the analysis of a broader scope of relevant pharmaceuticals, their metabolites and other relevant contaminants.

## Supplementary Information

Below is the link to the electronic supplementary material.

Supplementary file1 (DOCX 271 KB)

## Data Availability

The authors declare that the data supporting the findings of this study are available within the paper and its Supplementary Material file. Should any raw data files be needed in another format, they are available from the corresponding author upon reasonable request.
