# Supplementary material for: Application of QuEChERS extraction and LC–MS/MS for determination of pharmaceuticals in sewage sludges sampled across the Czech Republic
Source: Environ Sci Pollut Res Int. 2024 Nov 9;31(55):63946–58. doi: 10.1007/s11356-024-35508-8 (PMC11602849; doi:10.1007/s11356-024-35508-8)
Supplement: Supplementary file 1 — Supplementary file1 (DOCX 271 KB) [file 11356_2024_35508_MOESM1_ESM.docx]

**Supplementary material for:**

**Application of QuEChERS extraction and LC-MS/MS for determination of pharmaceuticals in sewage sludges sampled across the Czech Republic**

*Pavlína Landová^1, 2, *^, Ludmila Mravcová^1^, Šárka Poláková^2^, Petra Kosubová^2^*

^*^corresponding author: [xclandova@fch.vut.cz](mailto:xclandova@fch.vut.cz)

^1^Brno University of Technology, Faculty of Chemistry, Purkyňova 464/118, 612 00 Brno, Czech Republic

^2^Central Institute for Supervising and Testing in Agriculture (CISTA), Hroznová 63/2, 603 00 Brno, Czech Republic

**Supplementary material includes:**

[1. Description of the origin of samples 2](#_Toc164369632)

[2. Instrumental setup 3](#_Toc164369633)

[3. Composition of samples 4](#_Toc164369634)

[4. Extraction recoveries 6](#_Toc164369635)

[5. Influence of dilution on the reduction of matrix effect 8](#_Toc164369636)

[6. Literature review on the extraction and determination techniques and reported findings of target compounds in sewage sludge 10](#_Toc164369637)

[7. Determined concentrations of analysed pharmaceuticals in sewage sludge 12](#_Toc164369638)

[8. References 13](#_Toc164369639)

## Description of the origin of samples

Due to policy reasons, the anonymity of individual wastewater treatment plants must be preserved. However, the sampling dates and the regions of their locations are provided in Table S1. Capacity of individual WWTPs (expressed in equivalent inhabitants – EI) was variable. 2 facilities fell in the category of 50 to 2 000 EI, 10 were in the 2 001 to 10 000 EI range, 23 were in the 10 001 to 100 000 EI range, and 5 were above 100 001 EI.

**Table S1** Sampling dates and regions of the locations of WWTPs

| Sample no. | Sampling date | Region of the location of the WWTP |  |  | Sample no. | Sampling date | Region of the location of the WWTP |
| --- | --- | --- | --- | --- | --- | --- | --- |
| 1 | 24. 02. 2020 | Vysočina |  |  | **21** | 13. 05. 2020 | Karlovy Vary |
| 2 | 14. 05. 2020 | Vysočina |  |  | **22** | 11. 05. 2020 | Plzeň |
| 3 | 13. 05. 2020 | Vysočina |  |  | **23** | 23. 06. 2020 | South Moravian |
| 4 | 19. 05. 2020 | Pardubice |  |  | **24** | 23. 06. 2020 | South Moravian |
| 5 | 19. 05. 2020 | Pardubice |  |  | **25** | 23. 06. 2020 | Zlín |
| 6 | 13. 05. 2020 | Vysočina |  |  | **26** | 23. 06. 2020 | Olomouc |
| 7 | 13. 05. 2020 | Central Bohemian |  |  | **27** | 23. 06. 2020 | South Moravian |
| 8 | 13. 05. 2020 | Central Bohemian |  |  | **28** | 23. 06. 2020 | South Moravian |
| 9 | 14. 05. 2020 | Central Bohemian |  |  | **29** | 23. 06. 2020 | South Moravian |
| 10 | 14. 05. 2020 | Central Bohemian |  |  | **30** | 10. 06. 2020 | Vysočina |
| 11 | 14. 05. 2020 | Central Bohemian |  |  | **31** | 12. 06. 2020 | South Bohemian |
| 12 | 18. 05. 2020 | Zlín |  |  | **32** | 09. 06. 2020 | Vysočina |
| 13 | 18. 05. 2020 | Zlín |  |  | **33** | 09. 06. 2020 | South Bohemian |
| 14 | 19. 05. 2020 | Olomouc |  |  | **34** | 09. 06. 2020 | South Bohemian |
| 15 | 18. 05. 2020 | Zlín |  |  | **35** | 08. 06. 2020 | South Bohemian |
| 16 | 19. 05. 2020 | Olomouc |  |  | **36** | 22. 06. 2020 | Liberec |
| 17 | 19. 05. 2020 | Moravian-Silesian |  |  | **37** | 23. 06. 2020 | Ústí nad Labem |
| 18 | 13. 05. 2020 | Plzeň |  |  | **38** | 23. 06. 2020 | Liberec |
| 19 | 14. 05. 2020 | Plzeň |  |  | **39** | 23. 06. 2020 | Ústí nad Labem |
| 20 | 15. 05. 2020 | Plzeň |  |  | **40** | 23. 06. 2020 | Ústí nad Labem |

## Instrumental setup

**Table S2** Retention times, MRM transitions (including declustering potential (DP) and collision energies (CE)), and assigned internal standards to the target compounds

| ID | Rt (min) | Precursor mass (m/z) | DP (V) | Fragment mass 1 (m/z); CE (V) | Fragment mass 2 (m/z); CE (V) | Fragment mass 3 (m/z); CE (V) | Assigned internal standard |
| --- | --- | --- | --- | --- | --- | --- | --- |
| Atenolol | 1.42 | 267 | 25 | 190 (27) | 145 (35) |  | Atenolol-D7 |
| Trimethoprim | 1.93 | 291 | 86 | 230 (31) | 261 (35) |  | Trimethoprim-D9 |
| Acebutolol | 2.10 | 337 | 96 | 116 (30) | 98 (30) |  | Atenolol-D7 |
| Metoprolol | 2.23 | 268 | 30 | 116 (25) | 121 (29) | 77 (75) | Propranolol-D7 |
| Azithromycin | 2.31 | 375.2 | 60 | 591.3 (15) | 82.8 (40) |  | Azithromycin-D3 |
|  | 2.31 | 749.6 | 60 | 82.8 (85) |  |  |  |
| Sulfamethoxazole | 2.34 | 254 | 30 | 92 (37) | 156 (15) |  | Sulfamethoxazole-D4 |
| Bisoprolol | 2.53 | 326 | 96 | 116 (25) | 74 (47) |  | Propranolol-D7 |
| Propranolol | 2.62 | 260 | 86 | 116 (25) | 183 (27) |  | Propranolol-D7 |
| Citalopram | 2.68 | 325 | 91 | 109 (31) | 262 (27) |  | Fluoxetine-D5 |
| Fluoxetine | 2.78 | 310 | 71 | 44 (43) | 148 (13) |  | Fluoxetine-D5 |
| Carbamazepine 10,11-epoxide | 2.91 | 253 | 40 | 180 (37) | 236 (15) |  | Propranolol-D7 |
| Clarithromycin | 2.93 | 748.4 | 91 | 590.3 (27) | 158 (33) |  | Clarithromycin-D3 |
| Paroxetine | 2.94 | 330 | 80 | 192 (30) | 70 (60) |  | Sertraline-D3 |
| Norsertraline | 3.08 | 292 | 25 | 159 (31) | 275 (13) | 123 (65) | Sertraline-D3 |
| Carbamazepine | 3.09 | 237 | 31 | 194 (27) | 192 (31) |  | Carbamazepine-D10 |
| Sertraline | 3.10 | 306 | 30 | 275 (17) | 159 (35) |  | Sertraline-D3 |
| Atenolol-D7 | 1.41 | 274 | 25 | 190 (27) | 145 (35) |  |  |
| Trimethoprim-D9 | 1.91 | 300 | 86 | 264 (35) |  |  |  |
| Azithromycin-D3 | 2.31 | 377.25 | 60 | 82.8 (40) |  |  |  |
|  | 2.31 | 376.75 | 60 | 594.3 (15) |  |  |  |
|  | 2.31 | 753.6 | 60 | 82.8 (85) |  |  |  |
| Sulfamethoxazole-D4 | 2.34 | 258 | 30 | 96 (37) |  |  |  |
| Propranolol-D7 | 2.61 | 267 | 86 | 116 (25) |  |  |  |
| Fluoxetine-D5 | 2.77 | 315 | 71 | 44 (43) |  |  |  |
| Clarithromycin-D3 | 2.93 | 751.4 | 91 | 161 (34) | 593.3 (27) |  |  |
| Carbamazepine-D10 | 3.06 | 247 | 31 | 204 (29) |  |  |  |
| Sertraline-D3 | 3.10 | 309 | 30 | 275 (17) | 159 (35) |  |  |
|  | 3.10 | 311 | 30 | 277 (17) |  |  |  |

## Composition of samples

Analyses of elemental composition, organic content, and pH were carried out in the laboratories of our organisation (CISTA) following the standard procedures. Due to policy reasons, individual data cannot be provided. However, to illustrate the differences in the composition of individual samples, the heatmap was created, and it is available in Fig. S1. Nitrogen and organic content were analysed only in the fourteen samples (not analysed samples are indicated by blank spots within the heatmap).

| **Sample no.** | **Al** | **Ca** | **Fe** | **K** | **Mg** | **Na** | **Ʃ 13 other metals^1^** | **P** | **S** | **N** | **Org.^2^** | **pH** |
| --- | --- | --- | --- | --- | --- | --- | --- | --- | --- | --- | --- | --- |
|  | **mg kg^−1^ (d. w.)** | | | | | | | | | **% (d. w.)** | | **-** |
| **1** |  |  |  |  |  |  |  |  |  |  |  |  |
| **2** |  |  |  |  |  |  |  |  |  |  |  |  |
| **3** |  |  |  |  |  |  |  |  |  |  |  |  |
| **4** |  |  |  |  |  |  |  |  |  |  |  |  |
| **5** |  |  |  |  |  |  |  |  |  |  |  |  |
| **6** |  |  |  |  |  |  |  |  |  |  |  |  |
| **7** |  |  |  |  |  |  |  |  |  |  |  |  |
| **8** |  |  |  |  |  |  |  |  |  |  |  |  |
| **9** |  |  |  |  |  |  |  |  |  |  |  |  |
| **10** |  |  |  |  |  |  |  |  |  |  |  |  |
| **11** |  |  |  |  |  |  |  |  |  |  |  |  |
| **12** |  |  |  |  |  |  |  |  |  |  |  |  |
| **13** |  |  |  |  |  |  |  |  |  |  |  |  |
| **14** |  |  |  |  |  |  |  |  |  |  |  |  |
| **15** |  |  |  |  |  |  |  |  |  |  |  |  |
| **16** |  |  |  |  |  |  |  |  |  |  |  |  |
| **17** |  |  |  |  |  |  |  |  |  |  |  |  |
| **18** |  |  |  |  |  |  |  |  |  |  |  |  |
| **19** |  |  |  |  |  |  |  |  |  |  |  |  |
| **20** |  |  |  |  |  |  |  |  |  |  |  |  |
| **21** |  |  |  |  |  |  |  |  |  |  |  |  |
| **22** |  |  |  |  |  |  |  |  |  |  |  |  |
| **23** |  |  |  |  |  |  |  |  |  |  |  |  |
| **24** |  |  |  |  |  |  |  |  |  |  |  |  |
| **25** |  |  |  |  |  |  |  |  |  |  |  |  |
| **26** |  |  |  |  |  |  |  |  |  |  |  |  |
| **27** |  |  |  |  |  |  |  |  |  |  |  |  |
| **28** |  |  |  |  |  |  |  |  |  |  |  |  |
| **29** |  |  |  |  |  |  |  |  |  |  |  |  |
| **30** |  |  |  |  |  |  |  |  |  |  |  |  |
| **31** |  |  |  |  |  |  |  |  |  |  |  |  |
| **32** |  |  |  |  |  |  |  |  |  |  |  |  |
| **33** |  |  |  |  |  |  |  |  |  |  |  |  |
| **34** |  |  |  |  |  |  |  |  |  |  |  |  |
| **35** |  |  |  |  |  |  |  |  |  |  |  |  |
| **36** |  |  |  |  |  |  |  |  |  |  |  |  |
| **37** |  |  |  |  |  |  |  |  |  |  |  |  |
| **38** |  |  |  |  |  |  |  |  |  |  |  |  |
| **39** |  |  |  |  |  |  |  |  |  |  |  |  |
| **40** |  |  |  |  |  |  |  |  |  |  |  |  |

^1^ sum of the content of As, Be, Cd, Co, Cr, Cu, Mn, Mo, Ni, Pb, V, Zn, Hg

^2^ determined as loss on ignition at 550 °C

**Legend**

| **min** | >2 800 | >7 700 | >17 500 | >1 500 | >2 900 | >500 | >1 000 | >13 700 | >7 100 | >3.20 | >53.5 | >6.10 |
| --- | --- | --- | --- | --- | --- | --- | --- | --- | --- | --- | --- | --- |
|  | ≤9 640 | ≤17 580 | ≤31 720 | ≤3 160 | ≤4 440 | ≤940 | ≤1 640 | ≤20 920 | ≤9 760 | ≤3.90 | ≤58.2 | ≤6.64 |
|  | ≤16 480 | ≤27 460 | ≤45 940 | ≤4 820 | ≤5 980 | ≤1 380 | ≤2 280 | ≤28 140 | ≤12 420 | ≤4.60 | ≤62.9 | ≤7.18 |
|  | ≤23 320 | ≤37 340 | ≤60 160 | ≤6 480 | ≤7 520 | ≤1 820 | ≤2 920 | ≤35 360 | ≤15 080 | ≤5.30 | ≤67.6 | ≤7.72 |
|  | ≤30 160 | ≤47 220 | ≤74 380 | ≤8 140 | ≤9 060 | ≤2 260 | ≤3 560 | ≤42 580 | ≤17 740 | ≤6.00 | ≤72.3 | ≤8.26 |
| **max** | <37 000 | <57 100 | <88 600 | <9 800 | <10 600 | <2 700 | <4 200 | <49 800 | <20 400 | <6.70 | <77.0 | <8.80 |

**Fig. S1** Heatmap of the elemental composition and other properties of individual sludge samples

## Extraction recoveries

Individual results for recoveries for all analytes are provided in the following tables: Table S3 and Table S4. When concentrations in non-spiked samples were higher than the fortification level, recoveries were not calculated (indicated as n. a. in the tables).

**Table S3** Extraction recoveries (%) for all tested compounds in individual samples, fortification level 10 µg kg^−1^

| Sample no. | Azithromycin | Clarithromycin | Sulfamethoxazole | Trimethoprim | Carbamazepine | Carbamazepine 10,11-epoxide | Citalopram | Fluoxetine | Paroxetine | Sertraline | Norsertraline | Acebutolol | Atenolol | Bisoprolol | Metoprolol | Propranolol |
| --- | --- | --- | --- | --- | --- | --- | --- | --- | --- | --- | --- | --- | --- | --- | --- | --- |
| 4 | 70.7 | 102.7 | 86.1 | 68.6 | 98.0 | 94.1 | n. a.^1^ | 91.1 | 90.4 | n. a. | n. a. | 85.1 | 45.9 | 88.6 | 95.3 | 83.8 |
| 4 | 76.5 | 95.6 | 85.1 | 69.8 | 95.6 | 88.5 | n. a. | 89.1 | 84.7 | n. a. | n. a. | 92.0 | 44.7 | 90.0 | 90.7 | 86.7 |
| 23 | n. a. | 76.5 | 83.3 | 70.7 | n. a. | 93.0 | n. a. | 93.1 | 90.3 | n. a. | n. a. | 87.7 | 50.7 | 88.6 | n. a. | 85.4 |
| 23 | n. a. | 82.8 | 99.5 | 72.2 | n. a. | 94.7 | n. a. | 93.0 | 88.3 | n. a. | n. a. | 94.9 | 47.6 | 90.9 | n. a. | 88.0 |
| 30 | n. a. | 99.9 | 81.8 | 69.7 | n. a. | 94.2 | n. a. | 86.2 | 98.2 | n. a. | n. a. | 92.3 | 52.8 | 87.1 | n. a. | 83.6 |
| 30 | n. a. | 101.9 | 81.8 | 71.7 | n. a. | 101.4 | n. a. | 88.8 | 94.4 | n. a. | n. a. | 87.8 | 53.7 | 92.1 | n. a. | 89.1 |
| 32 | n. a. | n. a. | 86.8 | 81.0 | 96.2 | 92.3 | n. a. | 90.5 | 103.6 | n. a. | n. a. | 85.4 | 69.3 | 91.6 | 88.7 | 87.9 |

^1^ not assessed

**Table S4** Extraction recoveries (%) for all tested compounds in individual samples, fortification level 200 µg kg^−1^

| Sample no. | Azithromycin | Clarithromycin | Sulfamethoxazole | Trimethoprim | Carbamazepine | Carbamazepine 10,11-epoxide | Citalopram | Fluoxetine | Paroxetine | Sertraline | Norsertraline | Acebutolol | Atenolol | Bisoprolol | Metoprolol | Propranolol |
| --- | --- | --- | --- | --- | --- | --- | --- | --- | --- | --- | --- | --- | --- | --- | --- | --- |
| 1 | n. a. | n. a. | 101.3 | 81.8 | 101.6 | 96.2 | n. a. | 96.1 | 86.7 | n. a. | n. a. | 91.7 | 53.4 | 98.9 | 98.1 | 93.8 |
| 2 | n. a. | 103.1 | 108.9 | 83.0 | 100.7 | 98.9 | n. a. | 89.4 | 88.8 | n. a. | n. a. | 91.3 | 56.2 | 103.5 | 94.9 | 95.0 |
| 3 | 81.2 | 92.5 | 92.0 | 73.3 | 96.4 | 95.7 | n. a. | 90.7 | 76.3 | n. a. | 70.5 | 82.9 | 48.9 | 90.6 | 89.4 | 84.7 |
| 4 | 62.2 | 103.8 | 96.4 | 79.2 | 99.6 | 100.0 | 97.6 | 94.3 | 76.8 | 99.1 | 74.5 | 91.7 | 56.5 | 100.9 | 98.2 | 94.8 |
| 5 | 84.6 | 96.0 | 97.3 | 74.2 | 98.0 | 101.7 | n. a. | 93.6 | 77.2 | n. a. | n. a. | 85.7 | 49.9 | 95.2 | 96.0 | 87.8 |
| 6 | 83.4 | 100.9 | 91.8 | 71.8 | n. a. | 99.4 | n. a. | 96.2 | 75.0 | n. a. | n. a. | 86.9 | 48.1 | 97.2 | 95.1 | 88.4 |
| 7 | n. a. | 91.7 | 94.6 | 82.5 | 91.5 | 99.1 | n. a. | 93.5 | 79.8 | n. a. | n. a. | 86.7 | 55.7 | 95.5 | 88.7 | 89.4 |
| 8 | 89.2 | 115.5 | 103.6 | 77.6 | 99.9 | 96.7 | n. a. | 101.9 | 90.8 | n. a. | 73.4 | 88.5 | 51.0 | 94.3 | 94.4 | 93.0 |
| 9 | 83.5 | 74.4 | 84.9 | 69.9 | 83.0 | 93.1 | n. a. | 83.6 | 78.1 | n. a. | 70.3 | 78.5 | 47.9 | 88.0 | 87.6 | 82.9 |
| 10 | 78.5 | 98.2 | 87.5 | 75.0 | 92.5 | 94.8 | n. a. | 91.6 | 79.7 | n. a. | 76.2 | 87.5 | 52.2 | 89.8 | 90.1 | 85.9 |
| 11 | 67.9 | 107.4 | 83.6 | 71.2 | 97.8 | 87.2 | n. a. | 86.7 | 78.0 | n. a. | 75.5 | 79.8 | 46.3 | 85.6 | 84.7 | 79.7 |
| 12 | 71.1 | 95.5 | 84.4 | 72.1 | 94.7 | 95.3 | n. a. | 86.2 | 76.2 | n. a. | n. a. | 82.5 | 47.4 | 92.4 | 90.8 | 87.9 |
| 13 | n. a. | 98.1 | 95.1 | 77.6 | 96.4 | 92.9 | n. a. | 90.5 | 78.7 | n. a. | 73.2 | 84.2 | 51.9 | 93.7 | 91.4 | 86.4 |
| 14 | n. a. | 109.1 | 82.6 | 78.8 | 94.5 | 104.7 | n. a. | 96.7 | 95.6 | 92.0 | 69.6 | 89.3 | 53.2 | 101.0 | 98.4 | 96.6 |
| 15 | n. a. | 96.4 | 94.8 | 71.8 | 92.2 | 102.5 | n. a. | 90.5 | 78.0 | n. a. | n. a. | 85.0 | 48.2 | 98.4 | 97.7 | 93.6 |
| 16 | 79.0 | 89.2 | 100.6 | 78.6 | 93.8 | 104.2 | n. a. | 92.2 | 75.7 | n. a. | 68.9 | 87.0 | 52.8 | 97.8 | 97.2 | 90.8 |
| 17 | 83.4 | 96.7 | 98.7 | 75.6 | 94.9 | 95.4 | n. a. | 96.5 | 82.5 | n. a. | 72.4 | 90.0 | 53.5 | 95.2 | 96.0 | 85.2 |
| 18 | 83.3 | 87.0 | 90.7 | 74.2 | 97.7 | 100.5 | n. a. | 94.3 | 78.1 | n. a. | 76.2 | 87.8 | 51.9 | 97.2 | 98.4 | 93.4 |
| 19 | 93.1 | 89.3 | 93.7 | 82.7 | 100.4 | 101.1 | n. a. | 102.4 | 94.3 | n. a. | 81.7 | 91.5 | 54.2 | 101.6 | 99.1 | 97.7 |
| 20 | 81.8 | 97.6 | 85.3 | 77.3 | 95.9 | 99.7 | n. a. | 93.6 | 80.0 | n. a. | 68.3 | 88.1 | 52.6 | 92.9 | 94.5 | 88.0 |
| 21 | 93.9 | 92.2 | 95.8 | 86.4 | 103.2 | 105.8 | 104.0 | 99.8 | 90.4 | n. a. | 96.4 | 92.9 | 48.6 | 102.6 | 101.4 | 96.1 |
| 22 | 90.4 | 97.2 | 92.6 | 79.0 | 96.3 | 98.9 | 102.6 | 101.3 | 86.3 | n. a. | 85.3 | 96.0 | 55.0 | 96.3 | 96.3 | 92.4 |
| 23 | 81.0 | 94.3 | 99.9 | 73.4 | 98.4 | 90.3 | n. a. | 91.2 | 87.3 | n. a. | 93.5 | 90.2 | 50.9 | 89.8 | 94.5 | 84.7 |
| 24 | n. a. | 92.8 | 99.7 | 72.5 | 94.3 | 99.2 | n. a. | 87.1 | 86.8 | n. a. | 72.4 | 86.3 | 47.7 | 98.1 | 94.4 | 87.7 |
| 25 | 67.3 | 96.9 | 87.1 | 77.4 | 97.2 | 94.9 | n. a. | 96.3 | 75.7 | n. a. | 77.2 | 85.5 | 50.6 | 94.0 | 95.8 | 88.8 |
| 26 | n. a. | 106.7 | 100.6 | 74.5 | 97.9 | 87.1 | n. a. | 94.0 | 89.0 | 102.0 | 86.9 | 87.4 | 49.1 | 90.7 | 92.4 | 88.9 |
| 27 | n. a. | 81.4 | 88.3 | 74.3 | 93.0 | 90.6 | n. a. | 96.7 | 88.5 | n. a. | 75.7 | 87.3 | 47.7 | 91.9 | 95.1 | 91.0 |
| 28 | 86.6 | 92.1 | 102.0 | 80.9 | 99.6 | 95.5 | 101.8 | 95.7 | 87.3 | n. a. | 81.7 | 89.6 | 52.0 | 94.2 | 91.9 | 89.0 |
| 29 | 70.4 | 101.9 | 86.4 | 77.7 | 97.0 | 96.0 | n. a. | 89.4 | 86.8 | n. a. | 82.8 | 84.5 | 50.8 | 92.9 | 92.4 | 89.1 |
| 30 | 85.7 | 98.8 | 98.0 | 72.4 | 94.2 | 92.9 | 96.6 | 99.8 | 86.2 | 89.2 | 82.8 | 82.5 | 50.5 | 92.7 | 85.6 | 86.3 |
| 31 | 86.0 | 95.5 | 97.4 | 82.4 | 102.8 | 101.9 | 103.6 | 96.0 | 82.4 | n. a. | 66.8 | 87.2 | 51.5 | 103.4 | 98.8 | 97.3 |
| 32 | 93.8 | 107.9 | 105.3 | 83.1 | 102.3 | 96.1 | 98.3 | 100.1 | 89.9 | 89.5 | 77.9 | 93.2 | 55.7 | 98.1 | 95.2 | 93.5 |
| 33 | 92.8 | 103.2 | 98.4 | 77.1 | 104.5 | 103.4 | 104.7 | 105.5 | 85.0 | n. a. | n. a. | 90.4 | 48.9 | 99.1 | 98.5 | 94.8 |
| 34 | 84.2 | 99.8 | 100.2 | 76.3 | 96.7 | 99.8 | n. a. | 88.4 | 88.1 | n. a. | n. a. | 93.1 | 55.0 | 96.0 | 93.4 | 89.4 |
| 35 | 77.9 | 101.2 | 87.5 | 74.1 | 95.2 | 95.0 | n. a. | 94.6 | 82.8 | n. a. | n. a. | 86.3 | 50.2 | 94.6 | 90.8 | 87.1 |
| 36 | n. a. | 114.2 | 93.1 | 74.2 | 92.7 | 96.3 | n. a. | 96.1 | 83.8 | n. a. | n. a. | 85.1 | 48.4 | 91.9 | 91.8 | 88.9 |
| 37 | 59.7 | 103.0 | 92.0 | 64.3 | 95.9 | 87.0 | n. a. | 80.9 | 62.4 | 78.9 | 66.4 | 66.5 | 40.5 | 82.7 | n. a. | 61.0 |
| 38 | n. a. | 99.6 | 91.7 | 71.7 | 96.7 | 95.2 | n. a. | 87.9 | 80.6 | n. a. | n. a. | 79.3 | 45.5 | 93.8 | 91.2 | 82.9 |
| 39 | 70.5 | 98.3 | 83.9 | 67.7 | 86.4 | 87.2 | n. a. | 91.2 | 82.6 | n. a. | n. a. | 84.5 | 45.7 | 88.0 | 85.8 | 81.2 |
| 40 | 78.4 | 97.6 | 83.4 | 70.4 | 91.3 | 93.4 | n. a. | 90.7 | 88.9 | n. a. | n. a. | 84.9 | 47.6 | 95.0 | 96.5 | 89.1 |

^1^ not assessed

## Influence of dilution on the reduction of matrix effect

As we observed during the analysis of sludge samples, the variability of relative matrix effects (MEs) was higher among those analytes for which corresponding isotopically labelled internal standards (ILIS) were lacking. Our perspective for the future is to increase the number of target compounds, and the use of the respective ILIS for each compound is both economically and practically not feasible. Reducing matrix effects by developing a suitable cleanup procedure is difficult, especially when targeting multiple analytes with diverse chemical properties. In addition, the variable sample composition of sewage sludge may affect the cleanup process, thus reducing the repeatability of the procedure. Routinely applying a standard addition approach for quantification is also not practical. Since sludge usually contains many substances at different concentration levels, excessive additions would be required to cover the concentration range. As instrumental techniques are becoming more advanced and sensitive, the “dilute-and-shoot” approach is now more of an option.

 Two samples were selected to test the influence of dilution on the MEs. We assessed both quantification approaches (ESTD = external standard; ISTD = internal standard). Different dilutions were tested, from the original d2 approach (equal to 500 µL of sample extract filled up to 1000 µL) to d20 (equal to 50 µL of sample extract filled up to 1000 µL). The MEs were calculated as described in the manuscript. The results are shown in Figure S2.

The dilution was beneficial for all compounds by reducing MEs. However, excessive changes between calculated MEs were observed for bisoprolol when using ISTD calibration (assigned ISTD propranolol-D7). A comparison of the behaviour of bisoprolol and propranolol using ESTD shows that the dilution has a very different impact on the MEs of these two compounds. While the absolute MEs for bisoprolol are almost entirely reduced using further dilution, the same is not valid for propranolol. In particular, when analysing highly complex samples, such as sewage sludges, the use of internal standards which are not analyte-specific may be counterproductive. It may lead to additional errors in the results.

In conclusion, further dilution proved to be effective in the reduction of MEs. Quantification without internal standards can be performed using a dilution factor of 10 or 20. A combination of both ESTD and ISTD approaches would also be possible. However, further verification is necessary prior to routine application.

**Fig. S2** Influence of different dilutions on analytes matrix effects using ESTD calibration in (a) sample 17, and (b) sample 21; and using ISTD calibration in (c) sample 17 and (d) sample 21

(c)

(d)

(b)

(a)


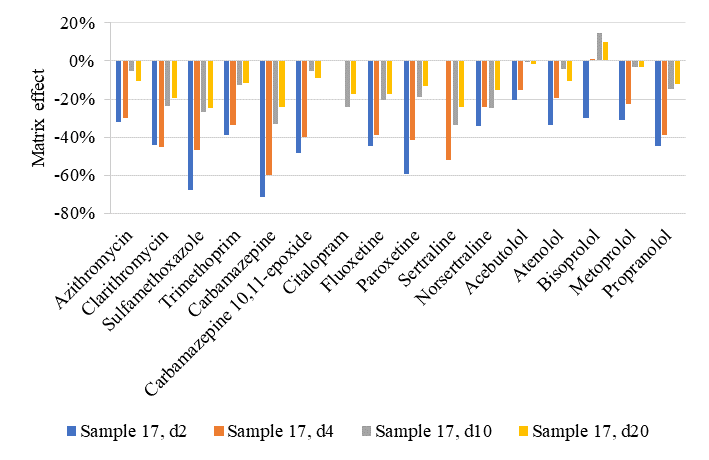

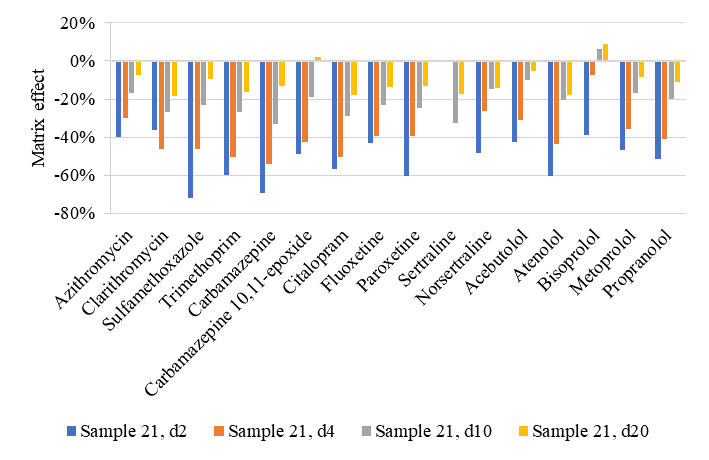

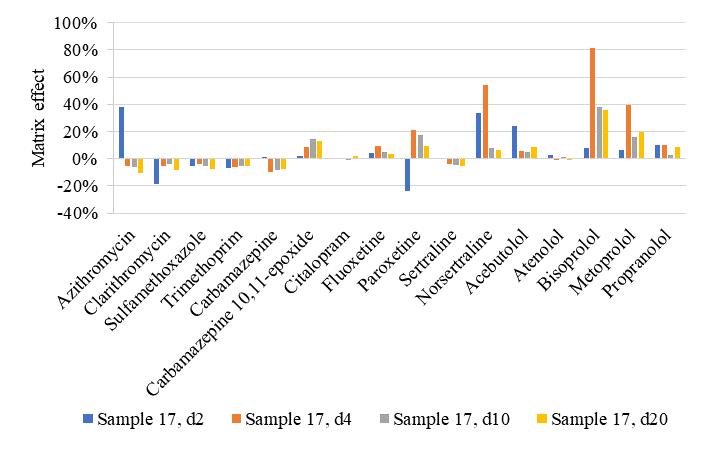

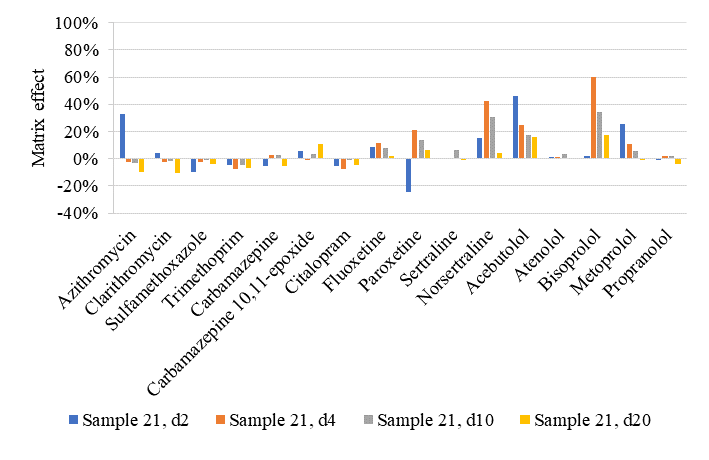


## Literature review on the extraction and determination techniques and reported findings of target compounds in sewage sludge

**Table S5** Literature review on the extraction and determination techniques, achieved recoveries, MDL and MQL, and the determined concentrations of selected pharmaceuticals in sewage sludge

|  | Extraction (cleanup) technique | Determination technique | Recovery %  (spiking level, µg kg^−1^) | MDL/MQL  (µg kg^−1^) | c (µg kg^−1^) | Sample origin | Reference |
| --- | --- | --- | --- | --- | --- | --- | --- |
| Azithromycin | PLE (SPE) | LC-MS/MS (TQ) | 81.1 (200) | –/17.9 | 38.8 | Spain | (Radjenović et al. 2009) |
|  | QuEChERS (D-SPE) | LC-MS (TOF) | 88/120/72 (250/1000/25000) | 16/49 | MDL–666 | France | (Peysson and Vulliet 2013) |
|  | USE (–) | LC-MS/MS (TQ) | 92 (40) | 6.5/21.6 | 60.8–267 | Greece | (Gago-Ferrero et al. 2015) |
| Clarithromycin | QuEChERS (D-SPE) | LC-MS (TOF) | 107/126 (250/1000) | 8/27 | MDL–MQL | France | (Peysson and Vulliet 2013) |
|  | USE (–) | LC-MS/MS (TQ) | 73 (40) | 3.3/11 | MQL–41.1 | Greece | (Gago-Ferrero et al. 2015) |
|  | ASE (SPE) | LC-MS/MS (TQ) | 84.3 (400) | 0.11/0.36 | 6.8–35.9 | Italy | (Riva et al. 2021) |
|  | USE (–) | LC-MS/MS (TQ) | 80/120 (333/1333) | 4/13.3 | 40 | Spain | (Pérez-Lemus et al. 2022) |
| Sulfamethoxazole | PLE (SPE) | LC-MS/MS (TQ) | 86.2 (200) | –/2.77 | 0.6 | Spain | (Radjenović et al. 2009) |
|  | QuEChERS (D-SPE) | LC-MS (TOF) | 91/98/124 (250/1000/25000) | 17/50 | MDL–MQL | France | (Peysson and Vulliet 2013) |
|  | USE (–) | LC-MS/MS (TQ) | 63 (40) | 4/12.2 | MDL | Greece | (Gago-Ferrero et al. 2015) |
|  | ASE (SPE) | LC-MS/MS (TQ) | 98.5 (400) | 0.55/1.85 | 7.1–13.4 | Italy | (Riva et al. 2021) |
|  | QuEChERS (D-SPE) | LC-MS (Orbitrap) | 78/77/82 (25/100/250) | 7.5/25 | MQL-10 | Greece | (Miserli et al. 2023) |
|  | USE (–) | LC-MS/MS (TQ) | 100/89 (333/1333) | 0.1/0.3 | 16 | Spain | (Pérez-Lemus et al. 2022) |
| Trimethoprim | PLE (SPE) | LC-MS/MS (TQ) | 29.3 (200) | –/2.97 | 10.5 | Spain | (Radjenović et al. 2009) |
|  | QuEChERS (D-SPE) | LC-MS (TOF) | 74/87 (250/1000) | 4/13 | MDL–13 | France | (Peysson and Vulliet 2013) |
|  | USE (–) | LC-MS/MS (TQ) | 83 (40) | 1/3.3 | MDL | Greece | (Gago-Ferrero et al. 2015) |
|  | USE (–) | LC-MS/MS (TQ) | 116/120 (333/1333) | 0.5/1.6 | <10 | Spain | (Pérez-Lemus et al. 2022) |
|  | QuEChERS (D-SPE) | LC-MS (Orbitrap) | 60/66/81 (25/100/250) | 1.9/6.2 | MQL–8.5 | Greece | (Miserli et al. 2023) |
| Carbamazepine | PLE (SPE) | LC-MS/MS (TQ) | 92.4 (10 – wet weight) | 0.17/0.58 | 258.1 | Canada | (Miao et al. 2005) |
|  | PLE (SPE) | LC-MS/MS (TQ) | 81.6 (200) | –/2.1 | 79.8 | Spain | (Radjenović et al. 2009) |
|  | QuEChERS (D-SPE) | LC-MS (TOF) | 91/130 (250/1000) | 5/15 | MDL–50 | France | (Peysson and Vulliet 2013) |
|  | USE (–) | LC-MS/MS (TQ) | 76 (40) | 4.4/14.7 | 18–113 | Greece | (Gago-Ferrero et al. 2015) |
|  | ASE (SPE) | LC-MS/MS (TQ) | 93.5 (400) | 0.09/0.31 | 19.7–23.7 | Italy | (Riva et al. 2021) |
|  | USE (–) | LC-MS/MS (TQ) | 83/106 (333/1333) | 1.4/4.8 | 36 | Spain | (Pérez-Lemus et al. 2022) |
|  | QuEChERS (D-SPE) | LC-MS (Orbitrap) | 74/86/88 (25/100/250) | 0.3/1.1 | 13.1–24.5 | Greece | (Miserli et al. 2023) |
| Carbamazepine 10,11-epoxide | PLE (SPE) | LC-MS/MS (TQ) | 87.7 (10 – wet weight) | 0.07/0.23 | MDL | Canada | (Miao et al. 2005) |
|  | QuEChERS (D-SPE) | LC-MS (TOF) | 89/105 (250/1000) | 49/147 | MDL | France | (Peysson and Vulliet 2013) |

**Table S5** Continued

|  | Extraction (cleanup) technique | Determination technique | Recovery %  (spiking level, µg kg^−1^) | MDL/MQL  (µg kg^−1^) | c (µg kg^−1^) | Sample origin | Reference |
| --- | --- | --- | --- | --- | --- | --- | --- |
| Citalopram | QuEChERS (D-SPE) | LC-MS (TOF) | 59/73 (250/1000) | 38/115 | MQL–313 | France | (Peysson and Vulliet 2013) |
|  | USE (–) | LC-MS/MS (TQ) | 73 (40) | 3.6/11.9 | 110–168 | Greece | (Gago-Ferrero et al. 2015) |
|  | QuEChERS (D-SPE) | LC-MS (Orbitrap) | 80/88/96 (25/100/250) | 0.6/2.1 | 14.5–111 | Greece | (Miserli et al. 2023) |
| Fluoxetine | PLE (SPE) | LC-MS/MS (TQ) | 30 (200) | –/3.9 | 122.7 | Spain | (Radjenović et al. 2009) |
|  | QuEChERS (D-SPE) | LC-MS (TOF) | 50/64/78 (250/1000/25000) | 16/47 | MDL–212 | France | (Peysson and Vulliet 2013) |
|  | USE (–) | LC-MS/MS (TQ) | 19 (40) | 5.3/17.5 | MDL–36.8 | Greece | (Gago-Ferrero et al. 2015) |
|  | QuEChERS (D-SPE) | LC-MS (Orbitrap) | 88/91/104 (25/100/250) | 1.9/6.2 | MQL–15.7 | Greece | (Miserli et al. 2023) |
| Paroxetine | PLE (SPE) | LC-MS/MS (TQ) | 66.8 (200) | –/1.74 | 40.7 | Spain | (Radjenović et al. 2009) |
|  | QuEChERS (D-SPE) | LC-MS (TOF) | 47/77 (250/1000) | 1/3 | MDL–89 | France | (Peysson and Vulliet 2013) |
|  | USE (–) | LC-MS/MS (TQ) | 22 (40) | 3.1/10.4 | MDL | Greece | (Gago-Ferrero et al. 2015) |
|  | ASE (SPE) | LC-MS/MS (TQ) | 141.2 (400) | 0.33/1.10 | 22.9–23.5 | Italy | (Riva et al. 2021) |
|  | QuEChERS (D-SPE) | LC-MS (Orbitrap) | 98/104/102 (25/100/250) | 1.1/3.6 | 5.5–23.9 | Greece | (Miserli et al. 2023) |
| Sertraline | QuEChERS (D-SPE) | LC-MS (TOF) | 43/101 (1000/25000) | 143/428 | MDL–3834 | France | (Peysson and Vulliet 2013) |
|  | USE (–) | LC-MS/MS (TQ) | 36 (40) | 2.7/8.9 | 20.1–108 | Greece | (Gago-Ferrero et al. 2015) |
|  | QuEChERS (D-SPE) | LC-MS (Orbitrap) | 74/87/89 (25/100/250) | 0.9/3.1 | 12.9–94.5 | Greece | (Miserli et al. 2023) |
| Norsertraline | USE (–) | LC-MS/MS (TQ) | 47 (40) | 5.1/16.8 | MDL | Greece | (Gago-Ferrero et al. 2015) |
|  | QuEChERS (D-SPE) | LC-MS (Orbitrap) | 79/80/96 (25/100/250) | 5.47/16.6 | 19.8–48.3 | Greece | (Miserli et al. 2023) |
| Acebutolol | No data |  |  |  |  |  |  |
| Atenolol | PLE (SPE) | LC-MS/MS (TQ) | 89.8 (200) | –/5.3 | 7 | Spain | (Radjenović et al. 2009) |
|  | QuEChERS (D-SPE) | LC-MS (TOF) | 45/71 (250/1000) | 3/10 | MDL | France | (Peysson and Vulliet 2013) |
|  | USE (–) | LC-MS/MS (TQ) | 94 (40) | 5.1/16.9 | MDL | Greece | (Gago-Ferrero et al. 2015) |
|  | ASE (SPE) | LC-MS/MS (TQ) | 89 (400) | 0.37/1.24 | 8.76–15 | Italy | (Riva et al. 2021) |
|  | QuEChERS (D-SPE) | LC-MS (Orbitrap) | 63/100/95 (25/100/250) | 0.7/2.5 | 11.2–30.7 | Greece | (Miserli et al. 2023) |
|  | USE (–) | LC-MS/MS (TQ) | 111/116 (333/1333) | 14.1/46.9 | 346 | Spain | (Pérez-Lemus et al. 2022) |
| Bisoprolol | PLE (SPE) | LC-MS/MS (TQ) | n. a. | n. a. | 11–12 | Germany | (Scheurer et al. 2010) |
| Metoprolol | PLE (SPE) | LC-MS/MS (TQ) | 70.2 (200) | –/1.14 | MDL | Spain | (Radjenović et al. 2009) |
|  | USE (–) | LC-MS/MS (TQ) | 93 (40) | 5.1/16.8 | MDL–16.2 | Greece | (Gago-Ferrero et al. 2015) |
| Propranolol | PLE (SPE) | LC-MS/MS (TQ) | 60.8 (200) | –/3.01 | 26.2 | Spain | (Radjenović et al. 2009) |
|  | QuEChERS (D-SPE) | LC-MS (TOF) | 79/57 (250/1000) | 3/10 | 82–849 | France | (Peysson and Vulliet 2013) |
|  | USE (–) | LC-MS/MS (TQ) | 53 (40) | 3.1/10.4 | MDL–24.1 | Greece | (Gago-Ferrero et al. 2015) |
|  | USE (–) | LC-MS/MS (TQ) | 110/77 (333/1333) | 17.8/59.4 | 135 | Spain | (Pérez-Lemus et al. 2022) |

## Determined concentrations of analysed pharmaceuticals in sewage sludge

Carbamazepine 10,11-epoxide, which was not detected in any sample, is excluded from the following results in Table S6.

**Table S6** Determined concentrations of pharmaceuticals in sludge in µg kg^−1^ d. w.

| Sample no. | Azithromycin | Clarithromycin | Sulfamethoxazole | Trimethoprim | Carbamazepine | Citalopram | Fluoxetine | Paroxetine | Sertraline | Norsertraline | Acebutolol | Atenolol | Bisoprolol | Metoprolol | Propranolol |
| --- | --- | --- | --- | --- | --- | --- | --- | --- | --- | --- | --- | --- | --- | --- | --- |
| 1 | 435.20 | 374.89 | <MDL | 61.49 | 35.18 | 529.81 | 34.71 | 7.24 | 687.95 | 352.06 | 16.31 | <MDL | 15.84 | 93.59 | 15.01 |
| 2 | 472.18 | 80.03 | <MDL | 38.82 | 138.39 | 977.13 | 51.24 | 26.29 | 1250.14 | 749.93 | 57.07 | 10.09 | 26.72 | 105.08 | 4.68 |
| 3 | 131.59 | 27.84 | <MDL | 1.39 | 79.72 | 345.52 | 26.03 | 11.29 | 841.01 | 180.97 | <4.0 | <MDL | 4.54 | 65.61 | 1.95 |
| 4 | <4.0 | 8.13 | <MDL | <0.5 | 5.31 | 110.69 | 3.09 | <MDL | 223.73 | 45.17 | <MDL | <MDL | <2.0 | 12.11 | <MDL |
| 5 | 151.31 | 51.72 | <MDL | 1.97 | 130.15 | 404.88 | 91.50 | 33.41 | 850.52 | 536.02 | 12.27 | <4.0 | 10.20 | 194.74 | 7.92 |
| 6 | 184.85 | 10.40 | <MDL | 1.16 | 215.58 | 553.66 | 10.49 | 10.87 | 741.08 | 273.42 | 9.65 | ND | 17.58 | 131.91 | 6.09 |
| 7 | 256.11 | 91.34 | <MDL | 82.38 | 67.55 | 429.21 | 11.97 | 11.68 | 442.29 | 291.19 | 33.15 | 14.83 | 16.12 | 51.45 | 2.22 |
| 8 | 198.74 | 20.19 | <9.0 | 6.17 | 89.24 | 463.30 | 14.19 | <4.0 | 498.71 | 114.19 | 8.87 | 14.24 | 10.58 | 54.68 | 2.46 |
| 9 | 177.68 | 56.49 | <MDL | 4.98 | 29.35 | 584.55 | 18.16 | <4.0 | 421.66 | 134.24 | <4.0 | 8.58 | 14.47 | 79.47 | 1.41 |
| 10 | 88.72 | 5.79 | <MDL | 0.81 | 35.42 | 366.21 | 11.63 | <4.0 | 493.98 | 145.15 | <4.0 | <MDL | 6.91 | 26.91 | 8.44 |
| 11 | 58.30 | 7.32 | <MDL | 1.22 | 44.88 | 365.26 | 13.41 | <4.0 | 586.78 | 167.23 | <4.0 | <MDL | 13.78 | 47.13 | 7.25 |
| 12 | 93.12 | 10.05 | <MDL | <0.5 | 51.51 | 372.87 | 13.20 | 39.48 | 491.58 | 257.40 | 45.98 | <MDL | 14.24 | 46.75 | 3.06 |
| 13 | 259.00 | 15.15 | <MDL | 1.34 | 35.30 | 354.89 | 24.34 | 36.32 | 418.10 | 192.32 | 22.96 | <4.0 | 17.57 | 99.12 | 5.18 |
| 14 | 644.39 | 61.86 | <MDL | 9.86 | 27.05 | 372.00 | 28.18 | 20.01 | 226.02 | 70.40 | 47.64 | 11.54 | 28.86 | 148.71 | 5.60 |
| 15 | 268.94 | 10.07 | <MDL | 1.32 | 94.68 | 397.90 | 15.73 | 22.32 | 672.87 | 335.16 | 12.61 | <4.0 | 10.99 | 50.27 | 2.95 |
| 16 | 84.77 | 5.40 | <MDL | 0.81 | 27.70 | 531.82 | 19.42 | 14.24 | 527.74 | 129.45 | 15.51 | <MDL | 12.77 | 67.08 | 4.11 |
| 17 | 142.27 | 89.99 | <MDL | 2.03 | 114.18 | 404.39 | 14.13 | 20.49 | 342.93 | 137.64 | 19.43 | 9.64 | 16.17 | 78.35 | 1.45 |
| 18 | 100.74 | < 2.0 | <MDL | <0.5 | 28.18 | 240.47 | 7.01 | <MDL | 436.40 | 201.41 | <4.0 | <MDL | 7.42 | 68.67 | 3.81 |
| 19 | 112.84 | 20.91 | <MDL | 61.95 | 21.78 | 259.29 | 17.66 | <4.0 | 375.36 | 189.00 | 37.70 | 25.85 | 10.04 | 104.98 | <MDL |
| 20 | 95.30 | 137.32 | <MDL | <0.5 | 48.25 | 255.68 | 11.65 | 12.21 | 485.72 | 135.06 | 10.06 | 4.25 | 10.09 | 89.56 | 4.32 |
| 21 | 204.39 | 124.99 | <9.0 | 62.10 | 12.51 | 155.17 | 8.88 | <4.0 | 575.62 | 205.06 | 13.10 | <4.0 | 4.24 | 50.74 | 1.62 |
| 22 | 204.59 | 30.70 | <9.0 | 20.57 | 10.62 | 169.62 | 8.36 | <4.0 | 467.47 | 86.18 | 9.39 | <MDL | <2.0 | 22.84 | <MDL |
| 23 | 98.04 | 9.03 | <MDL | 1.17 | 115.06 | 388.18 | 10.04 | <4.0 | 454.43 | 157.98 | 6.63 | <MDL | 8.82 | 77.96 | 2.83 |
| 24 | 352.39 | 19.61 | <MDL | 2.34 | 32.30 | 315.21 | 7.89 | <MDL | 321.50 | 62.18 | 8.33 | <MDL | 5.98 | 48.32 | 4.38 |
| 25 | 39.39 | 2.24 | <MDL | <0.5 | 50.13 | 347.46 | 7.79 | <4.0 | 300.69 | 119.00 | <4.0 | <MDL | 7.24 | 38.30 | 2.26 |
| 26 | 266.22 | 7.05 | <MDL | 1.36 | 49.52 | 534.92 | 11.97 | 23.47 | 292.71 | 155.18 | 48.70 | 4.93 | 17.20 | 182.11 | 12.33 |
| 27 | 244.12 | 29.43 | <MDL | 6.10 | 25.24 | 350.22 | 12.29 | 17.28 | 428.63 | 102.44 | 38.71 | 8.54 | 8.81 | 144.36 | 2.20 |
| 28 | 183.72 | 16.14 | <MDL | 0.78 | 38.76 | 219.49 | 13.97 | <4.0 | 386.68 | 131.72 | 11.92 | <MDL | 11.97 | 55.64 | 2.06 |
| 29 | 46.77 | 4.27 | <MDL | <0.5 | 77.25 | 372.04 | 15.96 | 16.00 | 506.69 | 187.06 | 5.92 | <MDL | 6.82 | 77.35 | 5.10 |
| 30 | 66.00 | 6.57 | <MDL | 0.62 | 57.13 | 196.80 | 9.92 | <4.0 | 272.25 | 124.96 | 6.43 | <MDL | 3.03 | 41.13 | <MDL |
| 31 | 78.57 | 19.70 | <MDL | <0.5 | 16.04 | 191.47 | 12.31 | 5.11 | 343.83 | 112.45 | 5.56 | <MDL | 3.54 | 24.82 | 1.68 |
| 32 | 22.10 | 45.85 | <MDL | 1.65 | 7.32 | 65.31 | 3.06 | <MDL | 208.01 | 44.83 | <4.0 | 7.72 | ND | 5.10 | <MDL |
| 33 | 176.51 | 32.76 | <MDL | 11.59 | 34.50 | 200.98 | 17.20 | 25.79 | 689.93 | 229.12 | 30.73 | 8.25 | 16.79 | 42.05 | <1.0 |
| 34 | 188.16 | 60.64 | <MDL | 8.44 | 107.80 | 434.02 | 21.44 | 28.40 | 1127.48 | 480.15 | 60.93 | 10.54 | 21.04 | 86.16 | 12.97 |
| 35 | 116.28 | 8.99 | <MDL | 0.67 | 41.07 | 322.39 | 30.64 | 20.71 | 608.74 | 233.52 | 7.55 | <4.0 | 14.15 | 99.36 | 6.27 |
| 36 | 380.09 | 38.65 | <MDL | 6.70 | 57.04 | 619.72 | 12.73 | 19.18 | 565.63 | 217.14 | 80.95 | 7.02 | 30.70 | 125.23 | <MDL |
| 37 | 183.68 | 61.56 | <MDL | 95.51 | 194.25 | 268.78 | 23.84 | 7.03 | 163.38 | 95.56 | 63.93 | 46.57 | 55.90 | 596.14 | 8.97 |
| 38 | 327.33 | 11.60 | <MDL | 1.87 | 43.90 | 915.87 | 15.52 | 21.21 | 703.74 | 255.90 | 9.27 | <4.0 | 10.32 | 104.14 | 8.57 |
| 39 | 129.73 | 5.01 | <MDL | <0.5 | 59.37 | 377.00 | 26.68 | 17.92 | 644.87 | 307.92 | 14.19 | <4.0 | 19.09 | 98.91 | 4.30 |
| 40 | 136.58 | 4.46 | <MDL | <0.5 | 74.55 | 531.97 | 50.76 | 6.25 | 764.82 | 249.62 | 5.05 | <4.0 | 11.83 | 85.92 | 5.70 |

## References

Gago-Ferrero P, Borova V, Dasenaki ME, Τhomaidis ΝS (2015) Simultaneous determination of 148 pharmaceuticals and illicit drugs in sewage sludge based on ultrasound-assisted extraction and liquid chromatography–tandem mass spectrometry. Anal Bioanal Chem 407:4287–4297. https://doi.org/10.1007/s00216-015-8540-6

Miao XS, Yang JJ, Metcalfe CD (2005) Carbamazepine and its metabolites in wastewater and in biosolids in a municipal wastewater treatment plant. Environ Sci Technol 39:7469–7475. https://doi.org/10.1021/es050261e

Miserli K, Kosma C, Konstantinou I (2023) Determination of pharmaceuticals and metabolites in sludge and hydrochar after hydrothermal carbonization using sonication—QuEChERS extraction method and UHPLC LTQ/Orbitrap MS. Environ Sci Pollut Res 30:1686–1703. https://doi.org/10.1007/s11356-022-22215-5

Pérez-Lemus N, López-Serna R, Pérez-Elvira SI, Barrado E (2022) Analysis of 60 pharmaceuticals and personal care products in sewage sludge by ultra-high performance liquid chromatography and tandem mass spectroscopy. Microchem J 175:107148. https://doi.org/10.1016/j.microc.2021.107148

Peysson W, Vulliet E (2013) Determination of 136 pharmaceuticals and hormones in sewage sludge using quick, easy, cheap, effective, rugged and safe extraction followed by analysis with liquid chromatography-time-of-flight-mass spectrometry. J Chromatogr A 1290:46–61. https://doi.org/10.1016/j.chroma.2013.03.057

Radjenović J, Jelić A, Petrović M, Barceló D (2009) Determination of pharmaceuticals in sewage sludge by pressurized liquid extraction (PLE) coupled to liquid chromatography-tandem mass spectrometry (LC-MS/MS). Anal Bioanal Chem 393:1685–1695. https://doi.org/10.1007/s00216-009-2604-4

Riva F, Zuccato E, Pacciani C, et al (2021) A multi-residue analytical method for extraction and analysis of pharmaceuticals and other selected emerging contaminants in sewage sludge. Anal Methods 13:526–535. https://doi.org/10.1039/d0ay02027c

Scheurer M, Ramil M, Metcalfe CD, et al (2010) The challenge of analyzing beta-blocker drugs in sludge and wastewater. Anal Bioanal Chem 396:845–856. https://doi.org/10.1007/s00216-009-3225-7
